# Effects of social and environmental contexts on multi-male mating and mixed paternity in socially monogamous female prairie voles

**DOI:** 10.1098/rsos.220298

**Published:** 2022-10-05

**Authors:** Marissa A. Rice, Sydney M. Galindez, Joshua T. Garner, Alexander G. Ophir

**Affiliations:** ^1^ Department of Psychology, Cornell University, Ithaca, NY 14853, USA; ^2^ Department of Integrative Biology, Oklahoma State University, Stillwater, OK 74078, USA

**Keywords:** female fecundity, *Microtus ochrogaster*, reproductive decision-making, social context, sex ratio

## Abstract

With whom and how often to mate are fundamental questions that impact individual reproductive success and the mating system. Relatively few studies have investigated female mating tactics compared with males. Here, we asked how differential access to mates influences the occurrence of mixed paternity and overall reproductive success in socially monogamous female prairie voles (*Microtus ochrogaster*). We created male- and female-biased sex ratios of prairie voles living in semi-natural outdoor enclosures. We ran paternity analyses to determine the identity and number of mating partners females had and the number of offspring produced. We found that 57.1% of females had litters fathered by two or more males when males outnumbered females, and 87.5% of females had litters with more than one father when females outnumbered males. However, the percentage of mixed paternity and the total number of embryos were not statistically different between social contexts. We determined that female fecundity (i.e. number of embryos) correlated with the number of male fathers in each litter across social contexts. Although our study did not support the hypothesis that social context directly influences female mating decisions, it did suggest that female multi-male mating might lead to increased fertilization success under semi-natural conditions.

## 1. Introduction

Mating behaviours often vary between and within species because the reproductive decisions that an individual must make are based on multiple factors. For example, the composition of the social environment can influence decisions on whether or not to mate with a potential suitor, to mate guard or to seek extra-pair copulations. Indeed, reproductive decision-making can be influenced by competition, access to resources, and many abiotic features of the environment [1]. The process of reproductive decision-making obviously has consequences on the mating behaviour in which individuals engage. Oddly, many studies of mating behaviour focus on males and pay relatively little attention to female mating decisions. One key exception to this trend is a narrow focus on the study of female choice [2–4]. However, the factors that influence female reproductive success clearly go beyond the question of which is the best possible mate available. Indeed, understanding the broader reproductive strategies that females employ should include which males females choose to mate with, but must also consider whether or not to mate with more than one male. Investigation of strategies that enable females to maximize reproductive success will provide a more thorough understanding of the factors and processes that guide reproductive decision-making.

Females are often the choosy sex, and this creates contexts in which females are gatekeepers of reproductive success [3]. However, the social dynamics among females have the potential to alter the ways individual females within a population express their gatekeeping strategy. The basic tenet of natural and sexual selection rests on the assumption that each individual attempts to maximize their own reproductive success [5,6]. Although there are general patterns that often emerge at the population level, the individual variation in how individuals express their efforts to maximally reproduce should be dynamic, plastic, and varied within certain limits. Despite the fact that it is rarely investigated, evidence has emerged demonstrating that multiple male mating is one way in which females might increase their individual reproductive success [7–9]. So long as variation in female reproductive success occurs, the choices and tactics that females take should be susceptible to contextual variation even if the variation in reproductive success among females is comparatively more stable than that of males [10].

An understanding of how female variation in reproductive decision-making impacts reproductive success can only be achieved after consideration of the sources of contextual variation. In very general terms, we believe one can divide contextual factors based on the scale of the context. Specifically, one can focus on global contexts, which include factors such as precipitation, temperature, resource distribution and other factors often associated with latitude, which all can influence reproductive decisions. Alternatively, local contexts encompass the more immediate social dynamics that emerge from population structure and social interactions. The global (or ecological) context of where individuals live geographically can have broad effects on mating behaviours, and might create constraints that limit all individuals, regardless of the local context. For example, birds in temperate zones have different mating behaviours and strategies from those in the tropics due to a difference in an abundance of resources [11,12]. Differences in altitude can also produce alternative conditions that impact reproductive decisions [13,14]. Geographical factors like these can determine the availability and distribution of resources, which have cascading effects on the decision-making that is made at the individual level. Local context, on the other hand, can influence female reproductive decisions that relate to the social environment, such as competition and access to mates. Intraspecific competition among females is typically thought to be determined by individual access to environmental reserves like food, water, space and shelter [15], an idea consistent with global context limitations on reproductive decision-making. However, females' reproductive decisions are also influenced by the access and availability of mating opportunities [16]. In fact, a variety of local social contexts can influence the likelihood of female multiple mating. For example, it has been argued that one female can more easily mate with multiple males when there are more reproductively available males relative to females, thereby creating a polyandrous mating system [17]. Conversely, one male can more easily monopolize many females in a polygynous context when there are more reproductively available females to males [17] thereby limiting female multiple mating. These theoretical examples demonstrate how the ratio of reproductively available males relative to females can impact reproductive decision-making and skew the instances of multiple mating. In socially monogamous species, where operational sex ratios are more even than in polyandry or polygyny [6,18], the social context has the potential to impact the decisions to engage in extra pair mating. However, it is unclear whether more males to females would lead to increased multi-male mating (e.g. because the availability of males is relatively higher and opportunity to mate multiply is therefore greater), or to fewer (e.g. because the pressure on males to mate guard and limit female extra pair mating is greater). Indeed, hypotheses such as these remain to be tested empirically.

Assessing the factors that influence female reproductive decision-making can be difficult under mating systems in which males and females demonstrate strong reproductive skew. This is because asymmetrical reproductive contexts reinforce particular tactics and thus limit the variation that can be expressed among individuals [19–21]. A focus on a mating system in which reproductive success is more equitable among males and females, such as social monogamy, can avoid this complication and offer an opportunity to measure flexibility in female multiple mating. Social monogamy is by far the most common form of monogamy, and encompasses a monogamous system in which mating partners engage in multiple mating in and out of a social pair, and where the operational sex ratios are relatively more balanced providing more equivalent access to potential mates [6,18]. Thus, manipulation of access to mates within a socially monogamous mating system should induce variation in the potential for female reproductive decision-making and reveal if the social context impacts female multi-male mating decisions.

The prairie vole is a socially monogamous rodent that is well suited for such manipulations, because it is a terrestrial socially monogamous species [22,23] for which we have a well descried understanding of its behavioural ecology [24,25], adapts well to experiments under contained semi-natural contexts [26,27], and for which paternity analyses are robust [26,28–30]. Notably, prairie voles are capable of flexibility and variation in mating tactics and reproductive decision-making [22,24,31]. Because they can be studied in the field in outdoor semi-natural enclosures, prairie voles offer an opportunity to manipulate the local context (via changing the access to mates), while holding the global context (spatial and physical environment) constant. In this study, we manipulated female access to potential mates in semi-natural field enclosures to test the impact of skewed sex ratio on female reproductive success. To this end, we created two sex ratios, each with 12 females to ensure the total number of females per unit space would be the same in each condition. A male-biased sex ratio was established with 18 males to 12 females, and a female-biased sex ratio was established with 8 males to 12 females, thus keeping the total number of females per enclosure constant. We hypothesized that differential access to mates would alter the occurrences of female multi-male mating, confirmed with paternity analysis. As mentioned above, it is unclear how reproductive skew might influence the behavioural decisions made by females in terms of multi-male mating (i.e. would a female-biased context or a male-biased context produce more female multi-male mating?).

## 2. Material and methods

### 2.1. Subjects

Prairie voles used in this experiment were taken from our prairie vole laboratory breeding colony and were direct decedents of 15 unrelated breeding pairs of either F1 or wild-caught prairie voles that were trapped in Urbana-Champaign, IL, USA. All animals were kept on a 14 : 10 h light : dark cycle and housed in polycarbonate cages (28 × 18 × 13 cm). Temperature and humidity were maintained at 21°C ±1°C and 60% ±2%. Animals had ad libitum access to Rodent Chow (Harland Teklad, Madison, WI, USA) and water, and were provided nestlets for warmth and enrichment.

In total, we used 26 males and 24 females, split into two groups that each were entirely composed of unrelated individuals. Animals serving in this experiment were weaned from parents at postnatal day 21, and then co-housed with their same-sex siblings until they reached sexually maturity (60–90 days).

All animals were ear tagged and tail-clipped prior to introduction to semi-natural enclosures (see below) for identification purposes. Standard small mammal ear tags (S. Roestenburg, Riverton, UT, USA) uniquely identified each animal. We used a standard procedure to affix the aluminium ear tags (approx. 3 mm) by piercing the pinna of the outer ear with a pointed end of the tag and securing the tag by threading the point through a hole in the back and folding the point flat. Ear tag weight is negligible, discomfort during piercing is minimal and transient, and behaviour is not observably altered by such ear tagging. Anticipating that some males might die before recovery, we ensured that all males were genotyped by collecting a tail clipping prior to introducing the animals into the enclosures. To minimize discomfort, we applied a topical anaesthetic (bupivacaine) to the end of the tail. Using surgical scissors, we took a 2–3 mm clip of tissue from the tip of the tail, which was placed in 70% alcohol and frozen at −20°C. Wounds were cleaned and pressure was applied immediately to ensure no bleeding before returning an animal to its home cage. Tail clips produce brief and transient discomfort, and all animals resumed normal behaviour within minutes of the procedure. All procedures were approved by the Institutional Animal Care and Use Committee (IACUC: AS096) of Oklahoma State University (the institution where the fieldwork was conducted) and consistent with guidelines for the ethical use of animals in research.

### 2.2. Semi-natural fieldwork

This study was performed in semi-natural field enclosures located within the natural distribution of prairie vole habitat in Stillwater, Oklahoma. The two enclosures were adjacent to each other and each measured 40 × 20 m. They were constructed of aluminium walls and powder-coated steel tube frames. The walls extended 60 cm above and below ground, preventing subjects from escaping the enclosures, and preventing any other animals from entering the enclosures. Each of the enclosures was made up of the same type of soil and vegetation, which were suitable natural prairie vole habitat. Specifically, this vegetation consisted of dicots and mixed pasture grasses such as brome, fescue and rye.

Female subjects were randomly assigned to either a male- or female-biased sex ratio condition as described in Rice *et al*. [27]. Briefly, 12 females were put into each treatment, and the number of females was kept constant to ensure the total number of females per unit space would be the same in each condition. To create different social contexts, we varied the number of males in each condition. The sex ratio was 18 males to 12 females in the male-biased condition, and 8 males to 12 females in the female-biased condition.

Females were released into the field enclosures 1 day before males to ensure that females had the opportunity to explore the field enclosures and find suitable nesting sites without the influence of males. All animals were allowed to live freely for a four-week period, beginning the day after males were introduced to the enclosed area. After four weeks had passed, we used traditional Sherman and Fitch traps baited with sunflower seeds and oats to recapture the animals. Trapping occurred daily for 10 consecutive days, in which traps were set at dusk and checked at dawn. During the day, baited traps were set to remain open to encourage visitation and avoid capturing animals during the peak heat of the day. After five continuous days where no additional animals were recovered, we assumed untrapped animals had perished. Of the 50 animals (26 male; 24 female) in this study, all but six (four male; two female) were recovered.

### 2.3. Paternity analysis

#### 2.3.1. Tissue collection, embryo harvesting and DNA extraction

Upon returning to the laboratory, females (*N* = 22) trapped from the field were euthanized by CO_2_ suffocation and embryos (*N* = 49) were directly collected from the mothers. Adults (males and females) were re-sampled for DNA by collecting a biopsy of leg muscle, which was stored in a micropipette tube submerged in 70% ethanol. Samples were stored at −20°C until DNA extraction. To harvest embryos, we extracted each fetus from the mothers' uterine horns, placed each embryo on a clean (DNA free) surface, removed the embryonic sac and placenta, placed them in 70% ethanol, and stored them at −20°C. Some females did not produce any litters, thus 15 of the 22 females recaptured from the field had embryos. Litter sizes ranged from one to six embryos and had a mean (±s.e.m.) of 3.27 ± 0.38. Samples of whole-body tissue were collected from embryos directly before DNA extraction. All tissue samples were thawed, and DNA was extracted following standard Qiagen DNEasy spin-column protocols (Qiagen Inc., Valencia, CA, USA).

We note that although animals were in the enclosures for up to four weeks, prairie vole gestation lasts about three weeks (between 20 and 24 days). Typically, animals do not mate within the first few days of being introduced to the enclosures. However, to ensure females had not given birth prior to trapping, we examined the uteri of females closely to look for embryonic scaring, and found no evidence of this in females that were not pregnant at trapping. We also never caught mothers with pups latched to nipples; prairie vole pups demonstrate ‘tenacious nipple attachment’ [32] and can be dragged by mothers while latched. Lastly, we also examined the nipples of females at trapping to see if they were enlarged (i.e. evidence of suckling), and found no evidence for this. From this, we inferred that non-pregnant females were nulliparous.

#### 2.3.2. Microsatellite loci

With mothers' identity known with 100% certainty, we sought to determine which males were responsible for fertilizing each embryo. To determine these relationships, we used six microsatellite loci (MOE2, MSMM3, MSMM5, AV13, MSMM2 and MSMM6) previously demonstrated to be polymorphic in prairie voles [33–35]. For details, refer to Ophir *et al*. [26]. Briefly, the microsatellite loci were amplified with fluorescently labelled primers by standard three-step polymerase chain reaction with annealing temperatures ranging from 52 to 58°C. Products were sized with ROX 400 size standard on an automated sequencer using Genescan software. The sizes were then confirmed by two independent observers who recorded the genotypes for all individuals. Lastly, using GENEPOP 4.7 [36] the populations in each field context were tested for Hardy–Weinberg equilibrium and linkage disequilibrium, and neither assumptions were violated.

#### 2.3.3. Paternity assignment

We imported the sizes of the microsatellite loci into the population genetics software package CERVUS 3.0 [37]. Simulations were run for 10 000 cycles, and we used a confidence interval of 95% when determining paternity. Using this software, we were able to determine the degree of paternity and possible mixed paternity in all litters. The software calculates the natural logarithm of the likelihood-odds ratio (LOD) score for each male, which is used to determine paternity. A more positive LOD demonstrates that a male is likely to be the father compared with other males in the population. Four of six loci was the minimum number of loci required for an individual to be included. For each embryo, the male with the highest LOD score was deemed the father as long as the individual was twice as likely as the second-best candidate. We interpreted confirmed mixed-sired litters as a proxy for multiple mating. Therefore, although we cannot rule out multiple mating that did not result in successful fertilization, our estimates reflect a minimum estimate of multi-male mating.

## 3. Results

**Figure 1.**
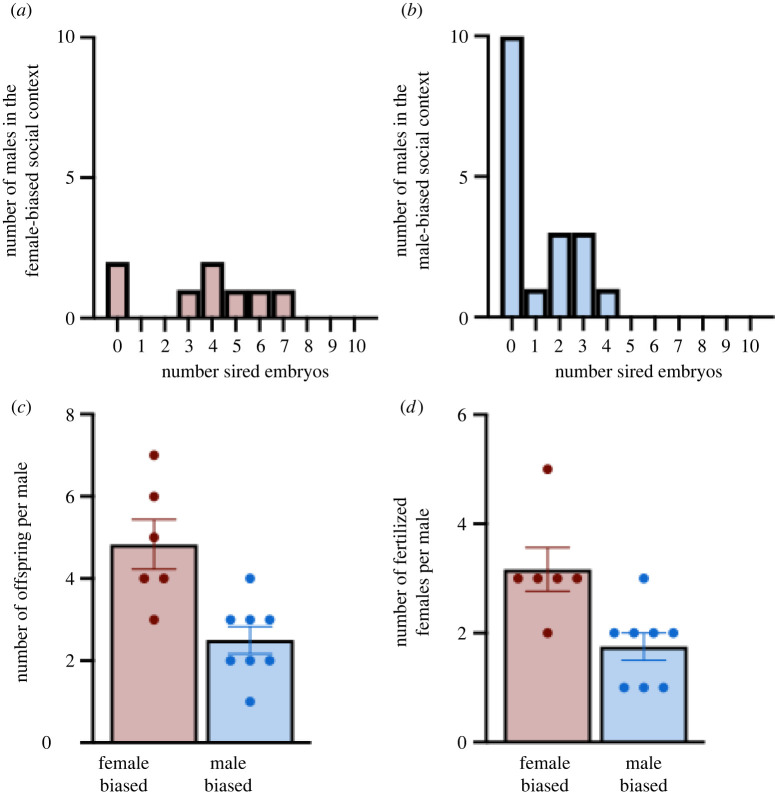
(*a*) Histogram presenting the number of males in the female-biased social context and the number of embryos that they fathered. (*b*) Histogram presenting the number of males in the male-biased social context and the number of embryos that they fathered. (*c*) The mean (±s.e.m.) number of pups that males in the female-biased (red) and male-biased (blue) social contexts sired. (*d*) The mean (±s.e.m.) number of females with which males in the female-biased (red) and male-biased (blue) social contexts sired offspring. Dots represent individual males in each group.

Our experiment was designed to manipulate female access to mates, while holding the number of females in each context constant. However, the number of males differed between social contexts as a result of this design. Thus, we found that 8 of 18 males (44.4%) from the male-biased context and 6 of 8 males (75%) from the female-biased social context sired offspring. This qualitative difference in fertilization success across males represents an expected consequence of the imbalance of males to females resulting from the reproductive skew (figure 1*a,b*). The skew in males to females also led to reproductively successful males in the female-biased context fathering nearly twice as many offspring *per capita* than those in the male-biased context (*t*_7.91_ = 3.410; *p* = 0.009; figure 1*c*). Likewise, males in the female-biased context fertilized embryos with a greater number of females then males in the male-biased context (*t*_8.7_ = 2.996; *p* = 0.016; figure 1*d*). Moreover, of the males that fertilized embryos with a female, 5 of 8 males (62.5%) in the male-biased social context and 6 of 6 males (100%) in the female-biased social context produced offspring with more than one female. Such results were expected considering that the female-biased context had more than twice the number of females per male, and caution should be taken when interpreting these results because the number of males was not comparable across contexts, unlike it was for females. Nevertheless, these results validate our experimental design and showed that the two social contexts created differences in mating availability and reproductive success for males. Thus, we were able to compare the impacts of differential access to males on female reproductive decision-making.

**Figure 2.**
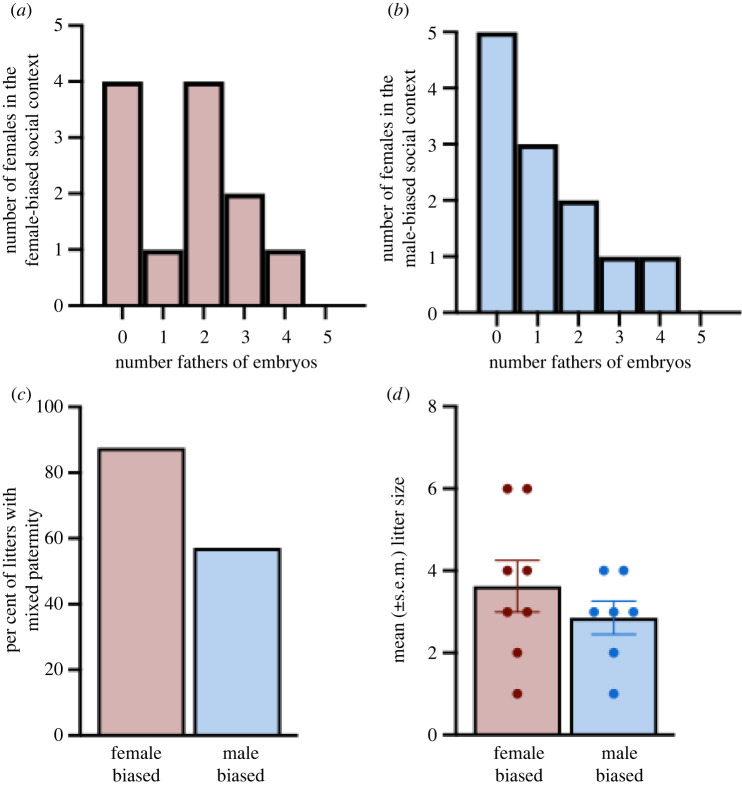
(*a*) Histogram presenting the number of females in the female-biased social context and the number of fathers that fertilized their embryos. (*b*) Histogram presenting the number of females in the male-biased social context and the number of fathers that fertilized their embryos. (*c*) The percentage of litters containing pups sired by multiple males in the female-biased (red) and male-biased (blue) social contexts. (*d*) The mean (±s.e.m.) number of pups per litter for females in the female-biased (red) and male-biased (blue) social contexts. Dots represent litter size for individual females in each group.

The goal of this experiment was to ask if the different social contexts (male biased or female biased) yielded different proportions of multi-male mating. After trapping, we found that 7 of 12 females (58.3%) from the male-biased context and 8 of 12 females (66.67%) from the female-biased social context were pregnant (figure 2*a*,*b*). Moreover, 57.1% (4 of 7) of females in the male-biased enclosure had litters sired by two or more fathers, whereas 87.5% (7 of 8) of females in the female-biased enclosure had litters with more than one father (figure 2*c*). Despite a nearly 30% difference, the percentage of mixed paternity was not statistically different between social contexts (Fisher's exact test, *p* = 0.282).

Next, we compared the fecundity of females within each social context and found that the total number of fertilized embryos did not differ between females from the female- or male-biased enclosures (Student's *t*-test: *t*_11.69_ = −1.03; *p* = 0.32; figure 2*d*). Taken together, despite females from the female-biased social context having a nominally greater proportion of mates, females in each social context did not significantly differ in their number of mates per litter, and the number of offspring sired between social contexts was equitable.

**Figure 3.**
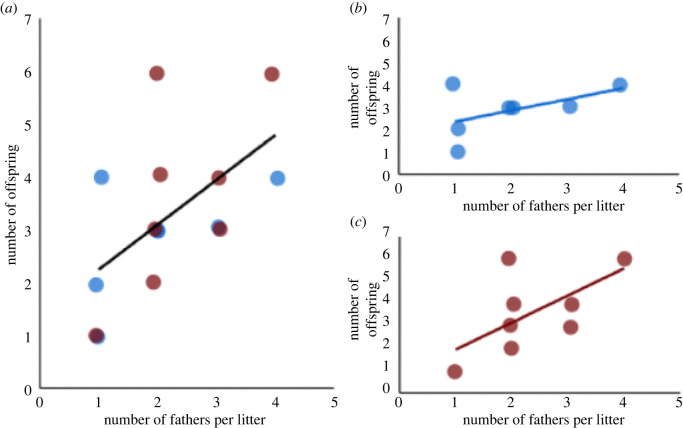
(*a*) The correlation between the number of pups per litter and the number of fathers corresponding to each litter, for females in both social contexts. The red dots correspond to females in the female-biased social context, and the blue dots correspond to females in the male-biased social context. (*b*,*c*) The correlation between the number of pups per litter and the number of fathers corresponding to each litter, for each female in the male-biased (*b*, blue) and female-biased (*c*, red) social contexts, respectively.

Because we saw high percentages of multiple mating within both social contexts, we determined whether female fecundity (i.e. total number of fertilized embryos) correlated with the number of male sires in each litter. A linear mixed model was used to analyse female reproductive success by comparing the number of offspring and number of fathers for male- and female-biased individuals. All the assumptions for the model were met. In the model, the number of offspring was the response variable, while the fixed effects were ‘number of fathers', ‘social context' and ‘number of fathers×social context'. The *F*-statistics reported are from Type III sum of squares tests. This analysis was consistent with the results above, showing that social context did not impact the total number of offspring females produced. Specifically, the number of fertilized embryos from the male- and female-biased enclosures did not differ (*F_3,14_* = 0.44; *p* = 0.52). Nonetheless, the analysis revealed that the number of fathers for a given litter was positively and significantly correlated with the number of offspring per litter for the female across all females (*F_3,14_* = 6.04; *p* = 0.03; figure 3*a*). This relationship between number of fathers and number of offspring was independent of social context, based on the absence of an interaction effect between ‘social context' and ‘number of fathers' (*F_3,14_* = 1.05; *p* = 0.33; figure 3*b*,*c*). Therefore, the general effect that female fecundity is positively and significantly correlated with the number of male partners (figure 3*a*), was independent of social context and the sex ratios that characterized them.

## 4. Discussion

Prairie voles have served as a very useful study species to better understand questions centred on monogamy and mating behaviour [24,38]. In addition to these classic studies of wild populations, prairie vole mating has also been studied in semi-natural field enclosures [26,29,39–43]. Relatively few studies, however, have focused solely on female prairie vole mating behaviour [44–47]. To enhance what is known about female reproductive decision-making, we sought to explore the mating decisions female prairie voles made in a semi-natural mating context (reflected by their choice to mate multiply or singly). In this experiment, our aim was to determine how differential access to mates would influence female mating behaviour. We created two distinct social contexts where the availability of mates was manipulated. We originally predicted that the social context (either male biased or female biased) would result in differential rates of multi-male mating; however, our results did not show statistical differences in the amount of female multi-male mating between social contexts. We acknowledge that due to our sample size, there is also a possibility of Type II error. Indeed, only 15 females produced pups out of our original sample of 24. To this end, we ran a *post hoc* power analysis to quantitatively determine the likelihood of a Type II error for the nearly 30% difference between mixed paternity litters in each social context, and found our power was 17.5% (*β* = 0.825). Thus, this explanation cannot entirely be ruled out when considering why we failed to see a difference in multiple mating between social contexts.

Despite our failure to find support for our original hypothesis, we did find that female reproductive success (i.e. number of embryos) increased as the number of male sires increased—a result that was independent of our manipulated contexts. This discovery raises important questions about the larger implications of how multi-male mating can impact female fecundity, and the ecological conditions that facilitate female multi-male mating as an optimal strategy.

### 4.1. The role of local social context in shaping behaviour

We originally hypothesized that local context (in this case, the access to mates) would impact reproductive decision-making for females. Our attempt to test this hypothesis, however, indicated that the manipulation of social context did not appear to impact mating behaviour or the rates of mixed-sire litters. This outcome is inconsistent with the theoretical support for the hypothesis that asymmetries in female access to mates impacts the rate of female multi-male mating [17]. Although it is still possible that the local social context alters the reproductive decision-making of females similar to how it alters cognitive processing [27], the access to mates based on sex ratios does not appear to drive such choices (but see below). Lack of support for this hypothesis raises the question of how extensively the local social environment impacts prairie vole behaviour, if at all?

The current study explored how mating decisions would differ by manipulating social context. We hypothesized that, like spatial cognition [27], the local differences in conspecific availability would also contribute to mating behaviour. This was partly based on the idea that a mating decision can be subdivided into many smaller decisions that are responsive to cognitive and social conditions [48]. However, although plasticity in mating tactics (i.e. the reaction to the immediate social environment [49]) are important for individual reproductive success, mating systems and the mating strategies that maintain them are established over an evolutionary timescale as a function of the predictable evolutionary pressures that shaped them in the first place [49]. It appears that in the case of female prairie voles' decisions to mate with multiple males, the forces from the social environment that shape mating strategies might supersede the forces that enable plasticity at the mating tactic level. The importance of the environment in determining tactical outcomes must account for the social environment, but also must consider the ecological forces that shape the evolution of mating systems [15]. On a relatively short timescale, local social contextual manipulations did not prove sufficiently robust to alter female mating tactics.

### 4.2. Does ecological, rather than local social, context shape female mating behaviour?

Previous estimates of female multi-male mating in this socially monogamous species have been quite low. For example, Ophir *et al*. [26] found that 16% of female prairie voles engaged in extra-pair mating (where 86% of females were bonded), and only 2 of 31 (6.5%) females produced mixed-sire litters under similar semi-natural conditions. By contrast, females (from both social contexts) demonstrated relatively high rates of multiple paternity, with more than half of all females (approx. 57–88%) engaging in multi-male mating. Relatedly, Solomon *et al*. [29] found that 5 of 9 (56%) wild-caught litters were sired by multiple males, a rate higher than Ophir *et al*. [26] but equal to or less than that of what is reported here. If local social context does not account for the rates of multiple mating, then what does? We believe that clues to answering this question can be found by accounting for the global (ecological) context.

We performed the field component of this study in Oklahoma, which is within the natural geographical distribution of prairie voles. However, most field or semi-natural enclosure studies with this species have occurred in Illinois, Indiana, Ohio or Tennessee [25,28,29,41,43,50]. Notably, some similar work has also taken place in Kansas, just north of our study site [28,51,52]. Prairie vole mating behaviour differs across their natural geographical distribution [50,53]. Populations of prairie voles in Kansas have demonstrated patterns of space use and home ranges that have suggested a weakened monogamous system [54]. Moreover, prairie voles from Kansas demonstrate higher percentages of mixed paternity when compared with populations in Illinois or Indiana [28,29]. Although it is more typical for female prairie voles to have litters with just one mate, the results from the Kansas studies are similar to our finding that females showed remarkably high levels of mixed-paternity litters. This raises the possibility that the global context (at the geographical or ecological level) is an important factor in influencing the decision for female prairie voles to mate with multiple males.

We ensured that the vegetation in enclosures matched the vegetation found in more northern areas like Illinois, Ohio or Indiana, suggesting that the vegetation, ground cover, or access to food resources was not the underlying cause of these between-study differences. However, we could not control for temperature, humidity and precipitation [55,56], leaving these variables as possible factors that influence female multi-male mating. Importantly, the arid nature of xeric geographical regions within the prairie vole distribution has been discussed as a possible cause for the variation in social monogamy among prairie vole populations [50,53] and could account for the high proportion of mixed-paternity litters we report. It remains to be seen if such ecological conditions might also account for the relationship between multi-male mating and litter size. Nevertheless, our study might represent an instance in which the features of the geographical context enabled the increased frequency of multi-male mating. It is very important to keep in mind, however, that many females did not mate multiply, indicating that not all females opt to mate with more than one male [46]. Taken together, our study highlights that the capacity for multi-male mating among most females is potentially associated with geographical/ecological variation, and less likely to be associated with local social contexts [6,28].

### 4.3. Female multiple mating might lead to increased fecundity

Although it was not our original intent to investigate the impact of multi-male mating on female reproductive success, our data demonstrated that female fecundity increased as a function of the number of males that fertilized their embryos. Thus, multi-male mating appears to be advantageous for female prairie voles, independent of social context. This result supports the hypothesis that multi-male mating might be a preferred mating tactic for females [57]. This conclusion is consistent with other studies in prairie voles [26,28,29,45–47] and a number of other taxonomic groups [7] that have shown that females mate multiply. Although the preference to bond and how it informs multiple mating remains unclear, these results provide an intriguing contrast to male prairie voles, which appear to prefer to form socially monogamous bonds [44]. Nevertheless, previous studies that have provided evidence of female prairie vole multi-male mating did not demonstrate a potential link between multi-male mating and fecundity [46], as the data from the current study seems to have done.

It is important to note that our study is unable to rule out the explanation that the chance to detect evidence of mixed-paternity litters should increase with an increasing litter size, and therefore data such as ours might not be a strong indicator of multi-male mating alone. Furthermore, it is not possible to monitor all mating events in the field, and thus it is plausible that some females mated multiply, but only produced offspring with one male. We acknowledge these inherent limitations, but we also point to the fact that socially monogamous females engage in multi-male mating [26,28,29,45–47] and the variation that exists therein as evidence that females presumably derive some advantage from mating with more than one male.

The most popular hypotheses for why females should mate multiply include that it increases the likelihood of successful fertilization [58,59], it increases offspring genetic diversity [58], or it creates paternity uncertainty [17,60–62]. Each of these theories offers a different perspective on the common theme of how females could maximize reproductive success. Despite these examples of how females benefit from multi-male mating, the question of why females engage in this behaviour flies in the face of sexual selection dogma, and has received relatively little attention compared with the more overwhelming belief that females choose and males compete to mate with multiple partners [5,63]. We believe that our data hint that multi-male mating might enhance fitness of female prairie voles, although targeted studies are necessary to test this hypothesis.

## 5. Conclusion

Stepping back, our study provides support for the conclusion that the ecological conditions found in warm dry environments (see above) might enable female multi-male mating as a result of challenges that the environmental context imposes. Clearly, additional work to test this hypothesis is needed. Nevertheless, our study has provided support for the conclusion that female reproductive decisions to mate with multiple males might lead to increased fertilization success under semi-natural ecological conditions, but that the availability of males does not appear to affect these decisions directly.

## Data Availability

The data are provided in electronic supplementary material [64].
